# Positive Toxicology Results Are Not Associated with Emergency Physicians’ Opioid Prescribing Behavior

**DOI:** 10.5811/westjem.2021.5.52378

**Published:** 2021-08-30

**Authors:** Jonathan B. Lee, Ghadi Ghanem, Soheil Saadat, Justin Yanuck, Brent Yeung, Bharath Chakravarthy, Ariana Nelson, Shalini Shah

**Affiliations:** * University of California, Irvine, Department of Emergency Medicine, Orange, California; † University of California, Irvine, Department of Anesthesiology & Perioperative Care, Orange, California

## Abstract

**Introduction:**

Given the general lack of literature on opioid and naloxone prescribing guidelines for patients with substance use disorder, we aimed to explore how a physician’s behavior and prescribing habits are altered by knowledge of the patient’s concomitant use of psychotropic compounds as evident on urine and serum toxicology screens.

**Methods:**

We conducted a retrospective chart review study at a tertiary, academic, Level I trauma center between November 2017–October 2018 that included 358 patients who were discharged from the emergency department (ED) with a diagnosis of fracture, dislocation, or amputation and received an opioid prescription upon discharge. We extracted urine and serum toxicology results, number and amount of prescription opioids upon discharge, and the presence of a naloxone script.

**Results:**

The study population was divided into five subgroups that included the following: negative urine and serum toxicology screen; depressants; stimulants; mixed; and no toxicology screens. When comparing the 103 patients in which toxicology screens were obtained to the 255 patients without toxicology screens, we found no statistically significant differences in the total prescribed morphine milligram equivalent (75.0 and 75.0, respectively) or in the number of pills prescribed (15.0 and 13.5, respectively). Notably, none of the 103 patients who had toxicology screens were prescribed naloxone upon discharge.

**Conclusion:**

Our study found no association between positive urine toxicology results for psychotropically active substances and the rates of opioid prescribing within a single-center, academic ED. Notably, none of the 103 patients who had toxicology screens were prescribed naloxone upon discharge. More research on the associations between illicit drug use, opioids, and naloxone prescriptions is necessary to help establish guidelines for high-risk patients.

## INTRODUCTION

In 2017 the opioid epidemic in the United States was declared a public health emergency.^1^ Opioid sales quadrupled from 1999 to 2012, possibly fueled by a marketing push by pharmaceutical companies, research indicating that opioids were not addictive, and statements by medical boards advocating for better treatment of pain.^2^–^5^ In 2018, physicians wrote 51.4 opioid prescriptions per 100 people. On a population level, this amounted to 12.8% of men and 17.2% of women in the US having at least one prescription filled for an opioid in 2018.^6^ That same year, with these high rates of prescribing, an average of 3.6% of Americans 12 and older self-reported prescription opioid abuse, resulting in 41 deaths per day.^6^,^7^ A major push to curtail opioid prescriptions has been initiated nationwide, yielding volumes of research and effective strategies to limit prescriptions.

Opioid prescriptions in the emergency department (ED) have been identified as a possible gateway for drug overuse or addiction. In a recent study of 53 patients who reported using heroin or nonmedical opioids, 59% of patients were first exposed to opioids by prescription, 29% of whom were first prescribed opioids in the ED.^8^ Furthermore, 12% of patients with acute pain who are prescribed opioids for the first time in the ED will continue to refill them after one year.^9^ The decision to prescribe opioids, and the quantity of opioids, can be subjective and may be influenced by the provider’s explicit and implicit biases. Studies have found that opioid prescription rates are dependent on the facility, physician, geographic location, and situational or workload factors.^10^–^13^ Other more implicit factors that have been identified may include a patient’s age, race, ethnicity, socioeconomic status, gender, insurance, clinical presentation, and physician’s judgment as to whether a patient may display drug-seeking behaviors.^14^–^19^

Physicians are often wary of prescribing opioids to patients who have a history of drug abuse or are taking illicit drugs that may cause an accidental overdose. However, this situation is further complicated when patients require opioids due to a major injury. Literature is sparse regarding guidelines on prescribing controlled medications to patients with suspected or confirmed illicit drug use.^20^ Previous literature has identified that individuals with alcohol, marijuana, hallucinogen, cocaine, stimulant, heroin, and sedative use disorders, as well as those with nicotine dependence, had a higher prevalence of prescription opioid use disorders.^21^ These individuals were also found to have used prescription opioids non-medically more often than those without substance use disorders, with an incidence rate ratio between 1.46 to 1.96.

Conversely, individuals misusing prescription opioids had much higher odds of using illicit drugs, including heroin, crystal methamphetamine, and cocaine.^22^ Given that nearly two-thirds of prescription opioid deaths co-occurred with cocaine, methamphetamine, or benzodiazepines, this presents a challenge to physicians who are prescribing opioids to patients with evidence of illicit substance use.^23^ Furthermore, a population-based cohort study of adolescents determined that illicit drug use is a risk factor for future opioid misuse in that population.^24^ In light of this evidence, it would be prudent for physicians to adjust their opioid prescribing habits, or co-prescribe an overdose-reversing agent such as naloxone to patients who require opioids but present with evidence of prior illicit substance use.

**Population Health Research Capsule**

What do we already know about this issue?

*Naloxone is an opioid antagonist designed to reverse overdose. Clinicians are encouraged to prescribe naloxone to patients who are at high risk for overdose*.

What was the research question?

*Does the presence of illicit drugs on drug screens have an association with naloxone or opioid prescriptions?*

What was the major finding of the study?

*The presence of illicit drugs did not have an association with rates of naloxone prescription or on the number of opioids prescribed*.

How does this improve population health?

*Clinicians should evaluate all protocolized labs ordered, as they may affect overall management. Naloxone should be y considered in the setting of high-risk, illicit drug use*.

With the recent legalization and increase in the use of cannabis and cannabinoid products including tetrahydrocannabinol (THC) and cannabidiol (CBD) in many states, it is important to consider the implications for opioid prescriptions. The most psychoactive component in the majority of cannabis products is THC, and it has been identified as playing a principal role in the analgesic effects of cannabis.^25^–^27^ To date, research bridging the years before and after medicinal and recreational cannabis legalization has demonstrated that the introduction of cannabis has either had no effect or decreased the quantity and dosage of opioid prescriptions.^28^–^31^ However, pre-clinical evidence is mixed regarding the opioid-sparing effects of THC. High quality clinical trials in humans are lacking, and results from the trials that have been conducted are mixed.^32^

Given the general lack of literature on opioid-prescribing guidelines for patients with substance use disorder, we aimed to explore how a physician’s behavior and opioid-prescribing habits may be altered by knowledge of the patient’s concomitant use of psychotropic compounds as evidenced on urine and serum toxicology screens. Additionally, our goal was to elucidate which patient populations are more likely to receive naloxone, and whether knowledge of recreational drug use through toxicology screens is associated with higher rates of naloxone prescriptions.

## METHODS

### Study Design and Setting

We conducted a retrospective chart review study in the ED of a tertiary, academic, Level I trauma center, between November 2017–October 2018.

### Selection of Participants

Patients 18 years of age and older who were discharged from the ED with a diagnosis of fracture, dislocation, or amputation and received an opioid prescription upon discharge were included in the study. We excluded from the analysis patients who were admitted to the hospital, transferred to another hospital, or not discharged with an opioid prescription. The study was reviewed and approved by the university’s institutional review board as an exempt category (Protocol number: HS#2018-4529). Patient informed consent was not applicable.

### Measurements

We obtained our data from the hospital’s health records database. We extracted the following information for each patient: age; gender; diagnosis (*International Classification of Diseases, 10^th^ Modification*); urine and serum toxicology results; prescription medication (name and dose); and quantity (number of tablets). For each patient we calculated a total prescribed milligram (mg) morphine equivalent (MME) by multiplying the prescribed amount (in mg) by potency of prescribed medication. The data collection was performed by a single abstractor, a pharmacist trained in using structured query language and the Observational Medical Outcomes Partnership. The abstractor was blinded to the study hypothesis.

### Patient Drug Use Classification

We divided the study population into five subgroups: patients with negative urine and serum toxicology screen; those who tested positive for depressants; stimulants; mixed; and no toxicology screens. A basic urine drug screen was used without confirmation testing. The drugs identified on the urine drug screen were amphetamines, barbiturates, cocaine, benzodiazepines, methadone, opiates, phencyclidine, THC, propoxyphene, and MDMA (3,4-methylenedioxy-methamphetamine). Alcohol was a quantitative test tested through serum. Depressants included patients who tested positive for alcohol, opiates, benzodiazepines, or methadone. Stimulants included patients with urine toxicology screens positive for methamphetamine or cocaine. The mixed subgroup contained urine or serum toxicology components from both the depressant and stimulant classes, as described above.

Given that THC has a complex pharmacology and its effects can vary from having depressant or stimulant properties depending on the dose, type, and individual user, any patient found to be THC positive was categorized as “mixed.” Because opiates and benzodiazepines are often used in the ED to treat painful conditions or for conscious sedation for fracture or dislocation reductions, patients with urine toxicology screens obtained after the ED administration of opiates or benzodiazepines were presumed negative for the substance, and the data was analyzed accordingly. Nine cases were presumed negative due to the patients having received an opioid or benzodiazepine prior to obtaining a urine sample for drug screen analysis: seven patients were presumed negative for opioids and recategorized from the depressant group to the negative group; one patient was presumed negative due to both benzodiazepine and opioid administration and recategorized from the depressant group to the negative group; and one patient was presumed negative for opioids and recategorized from the mixed group (due to presence of amphetamines) to the stimulant group. Of 103 patients who had a urine toxicology screen, eight had opioids that could not be explained by a prior opioid prescription or ED administration of an opioid. None of the patients in the stimulants group had active prescriptions for amphetamine-containing products such as dextroamphetamine for attention deficit hyperactivity disorder. All opioids and benzodiazepines identified to have been administered to these nine patients were confirmed by the institution’s lab to have been administered medications that are typically detected by the urine toxicology screen.

Furthermore, for trauma activations, the trauma service was actively involved in the care of patients including decisions on imaging, inpatient analgesics, and disposition. Once a patient is deemed stable for discharge from the ED by the trauma service, the rest of the patient’s care is up to the discretion of the emergency physician, which includes any and all medication prescriptions and ultimate disposition decisions. Lastly, as a supplementary analysis to look more specifically into potential associations with THC use we compared opioid prescriptions against three separate groups that included patients with negative toxicology screens for THC, patients with positive screens for THC, and patients without a toxicology screen.

### Analysis

Frequencies are reported as N (%). Continuous variables are reported as mean ± standard deviation (SD) or median (percentile 25 to percentile 75) if not distributed normally, as tested by the Kolmogorov-Smirnov test. Total prescribed MME was calculated by multiplication of medications’ MME by total mgs prescribed. We measured the amount of prescribed opioids by the number of pills (regardless of mg), or the volume of liquids (adjusted for concentration). We compared MME and prescribed amounts between subgroups of patients with urine toxicology by using the Kruskal-Wallis test. A *P*-value < 5% was considered statistically significant. We used SPSS Statistics 26 for Windows (IBM Corporation, Armonk, NY) for data analysis.

## RESULTS

### Characteristics of Study Subjects

**Figure 1:**
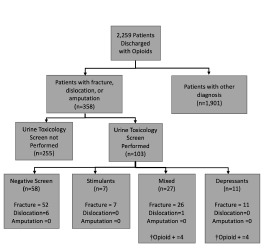
Study enrollment and exclusion November 2017–October 2018. Recruitment, enrollment, and exclusion of subjects. Flowchart indicates the study population and its categorization into the four groups: negative tox screen; positive for stimulants; “mixed”; and for depressants. In cases where the ED administered drugs known to affect the results of urine toxicology screens, patients were deemed presumptively negative for that substance and recategorized. † Opioid + refers to the number of patients who had opioids on urine toxicology screens that could not be explained by a prior opioid prescription or ED administration of an opioid.

From November 2017–October 2018, we retrospectively obtained 2259 unique records from visits associated with an opioid prescription upon discharge from the ED. Of this population of patients, 358 had a diagnosis of fracture (n = 335), dislocation(n =17), or amputation(n = 6). Within this group, 103 had urine toxicology screens. Of these 103 patients, 96 fractures. 7 dislocations, and 0 amputations were identified. Figure 1 displays overall study enrollment and exclusion. The mean age was 45.16 ±19.24, 72.8% (n = 75) were White, and 14.5% (n = 15) Asian. Medicaid patients comprised 34.0% (n = 35) of patients, 31.1% (n = 32) had commercial insurance, and 17.5% (n = 18) had Medicare (Table 1).

### Comparison of Morphine Milligram Equivalent Prescriptions

**Figure 2:**
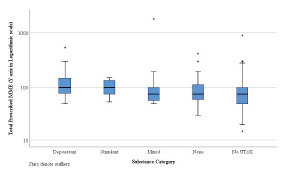
The median total morphine milligram equivalents across drug classes. There was no statistically significant difference in the median total morphine milligram equivalent between the five subgroups (p=0.074). * Represents outliers. *MME*, morphine milligram equivalent.

**Figure 3:**
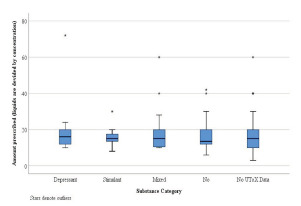
The median total amount of medications prescribed across drug classes. There was no statistically significant difference in the median number of pills between the five subgroups (P = 0.684). *represents outliers. *UToX*, urine toxicology screen.

The study population was divided into five subgroups that included the following: negative urine and serum toxicology screen (none); depressants; stimulants; mixed; and no toxicology screens. The median total MME for the five separate subgroups was as follows: none (75.0); depressant (100.0); stimulants (100.0); mixed (75.0); and no toxicology screens (75.0) (Figure 2). The median total number of pills for the five separate subgroups was as follows: none (13.5); depressant (16.0); stimulants (15.0); mixed (15.0); and no toxicology screen (15.0) (Figure 3). When comparing the 103 patients from whom toxicology screens were obtained to the 255 patients without toxicology screens, we found no statistically significant differences in the total prescribed MME (75.0 and 75.0, respectively) or in the number of pills prescribed (15.0 and 13.5, respectively). Notably, none of the 103 patients who had toxicology screens were prescribed naloxone upon discharge.

We also looked into whether the type of injury had any association with opioid prescriptions. Our data, shown in Table 2 below, indicates there was no statistically significant difference in total prescribed MME (*P* = 0.886) or amount of pills prescribed (*P* = 0.608) when comparing patients with fractures, dislocations, or amputations.

As a supplementary analysis we aimed to determine whether or not the presence of THC on urine toxicology screens was associated with an increase or decrease in the amount and total MME prescribed (Appendix, Table 1). The median total prescribed MME for patients with urine toxicology screens positive for THC was 87.5. The median (total MME) for patients with urine toxicology screens negative for THC was 75.0, and there was no statistically significant difference between the two groups (*P* = 0.991). The median total number of pills for patients with urine toxicology screens positive for THC was 15.0. The median total number of pills for patients with urine toxicology screens negative for THC was 15.0, and there was no statistically significant difference between the two groups (*P* = 0.740).

## DISCUSSION

At our Level I trauma center it is routine to obtain urine and serum toxicology screens for trauma activations. Most often, the results of these toxicology screens are not pertinent and will not significantly affect the patient’s disposition. However, previous reports have suggested that in some circumstances the urine drug screen is of utility in improving patient care by identifying patients who are at risk for diversion and mismanagement of controlled substances.^33^ Our results did not substantiate these reports. For context, providers in California must consult the Controlled Substance Utilization Review and Evaluation System (CURES), the state’s prescription drug monitoring program, prior to prescribing Schedules II–IV controlled substances for the first time and at least once every four months thereafter if the patient continues to use the controlled substances.^34^

However, if prescribed in the ED, providers do not have to consult CURES if the quantity of controlled substance does not exceed a nonrefillable seven-day supply. In fact, it is common practice to prescribe less than one week’s supply and to consult CURES only if the prescriber has suspicion of diversion, misuse, or abuse. For these reasons we suspect CURES reports likely had limited to no effect on prescribing habits.

A large-scale study based upon Medicaid States Drug Utilization Data found an associated decrease in the number of opioid prescriptions, dosages, and Medicaid spending in states that have legalized medical cannabis.^30^ A similar study found that in states that have legalized recreational marijuana, there was a notable decrease in opioid prescriptions of about 6.38%.^35^ Since then, several studies have failed to demonstrate similar findings in actual clinical practice, and many have actually found that cannabis use was associated with an increased risk of opioid use disorder and opioid misuse.^36^–^39^

In our study, we found no statistically significant difference in opioid prescriptions in terms of either total MME or number of pills prescribed between groups. Thus, we do not see that emergency physicians reduce or significantly change the quantity of prescribed opioids when urine toxicology screens are noted to be positive for THC. This was consistently true even when our study population was divided into different classes of toxicology results (stimulants, depressants, mixed, and negative results).There was also no difference in opioid prescriptions between these four separate groups. Thus, physician knowledge of prior drug use was not associated with a decrease in the total quantity (MME) of opioid prescriptions. This may be explained in part by the legal status of cannabis in the state of California and may portend an overall reduction in the stigma that was previously endured by patients who used cannabis medicinally or recreationally.

Another salient finding within this data was the absence of naloxone prescriptions for any patient in this study. In the state of California, Assembly Bill No. 2760 was passed on September 10, 2018, and took effect January 1 2019. This bill mandates that opioid prescribers must offer a prescription of naloxone hydrochloride when the prescription dosage is 90 MME or more per day, when an opioid is prescribed concurrently with a benzodiazepine, and when the patient is at increased risk for overdose, which includes patients with a history of overdose, patients with substance use disorder, or patients at risk for returning to a high dose of opioid medications.^40^ We collected the data for our study prior to the enactment of this law. However, it is prudent to recognize that even within this law, there is no clear mandate on prescribing naloxone based upon toxicology results that imply higher risk of illicit drug use, such as urine drug screens that are positive for both opioids and benzodiazepines. We also found that of the 103 patients who had toxicology screens performed, 57 (55.3%) were prescribed a total MME <90, and 46 (44.7%) were prescribed a total MME >90. Thus, had the law been in effect, 44.7% of these patients should have received a prescription for naloxone regardless of their drug screens, strictly due to the total MME prescribed. While this study was performed at an academic tertiary care center, if it were repeated at other community-based institutions, we could see similar patterns regarding the lack of naloxone prescriptions. Furthermore, we undertook this study in Orange County, California, a densely populated setting in Southern California that was ranked 17^th^ out of 58 counties in the state for rates of prescription opioid deaths and unintentional injuries. Drug overdose was the largest contributor and the number 1 cause of death in patients between the ages of 15–44 years old.^41^–^42^

One study that surveyed emergency providers at an academic, urban, Level I trauma center found that the factors most commonly influencing providers’ willingness to prescribe naloxone were the prevalence of prescribing these medications in their institution, or if there was a strong mortality benefit.^43^ Sixty-two percent of prescribers endorsed that lack of training was a barrier to prescribing, and 52% cited lack of knowledge as a barrier. Thus, it is pertinent that as a medical community, we focus on methods to improve research and education on naloxone so that prescribing can become a more common practice. Several initiatives have been developed and described in the literature aimed at improving naloxone prescription rates. Some examples include screening questionnaires for patients, pharmacy-led opioid overdose risk assessments, and multi-disciplinary teams with clinical nurse specialists for overdose education and naloxone distribution.

In one study a program was implemented within the electronic health record (EHR) system to search for keywords within nursing assessment notes to identify patients who were at high risk for opioid overdose. This then prompted the physician to consider naloxone prescriptions. Overall, the study found that since implementation of this integrated EHR programming, there was an associated increase in the rate of take-home naloxone prescriptions.^44^ Implementation of similar programming in EHRs could be used to flag patients with toxicology results positive for high-risk illicit drug use such as benzodiazepines, other opiates, and alcohol. These flagged patients could then trigger a prompt to consider prescribing naloxone if the clinician attempts to prescribe an opioid. Given that some states have implemented mandates requiring the prescription of naloxone when prescribing opioid regimens greater than 90 MME, an additional prompt from the EHR recommending naloxone in these situations may prove useful to ensure compliance with local laws and practice guidelines.^39^

## LIMITATIONS

Limitations of this study include the small sample size and retrospective nature of the review. Given that we conducted the study in a single-center, urban, academic, tertiary care center, we cannot extrapolate the results to community-based EDs or EDs in other states with their own state-specific laws regarding medical and recreational cannabis use. Furthermore, our patient population is unique to the region and cannot be generalized to the general US population.

In California, adult recreational use of cannabis was legalized in January 2018 under proposition 64.^45^ The study was conducted between November 2017–October 2018. Two months of data were collected prior to official legalization of adult-use recreational cannabis, and the remaining 10 months of data collection occurred after the January 1, 2018, start date of legal cannabis sales for recreational use. Given this, it is unclear how the new legislation might have affected physician perceptions of cannabis use. Future studies should expand the dataset to include data prior to legalization, and one full year after legalization to account for a washout period after which recreational use of cannabis was legalized.

Our database only included data for patients who received an opioid prescription. We could not analyze how drug screens may have affected disparities in prescribing opioids vs non-opioid analgesics to patients. Additionally. several confounding variables regarding opioid prescribing were not accounted for, such as the severity of injury, presence of multiple or prior injuries, race/ethnicity, payor type, prescriptions of non-opioids, verbally obtained social history, or comorbid conditions. Lastly, although use of urine toxicology screening provides us with an objective measure of drug use, there are limitations given these screens cannot tell us how frequently substances are being used or whether a positive screen means the patient is under the drug’s effects or it had been used in the past. Patients who are daily users of recreational drugs or actively intoxicated upon evaluation in the ED have different risk profiles than the occasional user.

## CONCLUSION

Our study at a single-center academic ED found no association between positive urine toxicology results for psychotropically active substances and significant difference in opioid prescriptions in terms of either total morphine milligram equivalent or the number of pills prescribed. The type of drug identified in urine toxicology screening did not have an association with the quantity of opioids prescribed or the rate of naloxone prescribing. Of note, our findings may act as a reminder that emergency physicians should evaluate all labs ordered by protocol-based order sets, as these often-overlooked tests may affect overall management and/or disposition. Further studies are needed to determine whether cannabis or illicit drug use influences the rate of opioid prescriptions, and how legalization of recreational use of cannabis has influenced physician prescribing habits and whether these findings can be generalized over larger populations and in states where cannabis has not been legalized. Overall, we observed a notable lack of naloxone prescriptions within a high-risk group of patients, underlining the need for further educational and/or institutional guidelines for naloxone prescribing.

## Supplementary Information



## Figures and Tables

**Table 1:** Baseline characteristics of patients with a diagnosis of fracture, dislocation, or amputation.

	Urine/serum toxicology obtained					

	No		Yes		Total	
Age (Mean ± SD)	44.7 ± 19.63		45.2 ± 19.24		44.8 ± 19.50	
Gender (N, %)						
Female	193	75.7%	71	68.9%	264	73.7%
Male	62	24.3%	32	31.1%	94	26.3%
Total	255	100.0%	103	100.0%	358	100.0%
Race (N, %)						
American Indian or Alaska Native	1	0.4%	0	0.0%	1	0.3%
Asian	35	13.7%	6	5.8%	41	11.5%
Black or African American	3	1.2%	2	1.9%	5	1.4%
Multi-race	1	0.4%	7	6.8%	8	2.2%
Native Hawaiian or other Pacific Islander	2	0.8%	0	0.0%	2	0.6%
Other race	32	12.5%	11	10.7%	43	12.0%
White	181	71.0%	77	74.8%	258	72.1%
Total	255	100.0%	103	100.0%	358	100.0%
Ethnicity (N, %)						
Hispanic or Latino	100	39.2%	43	41.7%	143	39.9%
Not Hispanic or Latino	153	60.0%	55	53.4%	208	58.1%
Unknown	2	0.8%	5	4.9%	7	2.0%
Total	255	100.0%	103	100.0%	358	100.0%
Insurance (N, %)						
Commercial	66	25.9%	42	40.8%	108	30.2%
Medicaid	106	41.6%	35	34.0%	141	39.4%
Medicare	43	16.9%	11	10.7%	54	15.1%
Other	24	9.4%	0	0.0%	24	6.7%
Other public	11	4.3%	14	13.6%	25	7.0%
Self-pay	5	2.0%	1	1.0%	6	1.7%
Total	255	100.0%	103	100.0%	358	100.0%

**Table 2:** Association between injury type and opioids prescribed.

	Injury type		

	Amputation	Dislocation	Fracture
Total prescribed MME			
Count	6	17	335
Minimum	50.0	25.0	15.0
Maximum	280.0	200.0	1800.0
Median	80.0	75.0	75.0
Mean	123.3	84.6	98.3
Standard deviation	95.64	47.09	120.06
Amount prescribed (liquids are divided by concentration)			
Minimum	10.0	5.0	3.0
Maximum	40.0	25.0	72.0
Median	16.0	15.0	15.0
Mean	20.0	13.6	15.9

There was no statistically significant difference in total prescribed MME (P = 0.886) or amount prescribed (P = 0.608) between fracture, dislocation, and amputation groups.

*MME*, morphine milligram equivalent.

## References

[1] US Department of Health and Human Services (2018). HHS Acting Secretary Declares Public Health Emergency to Address National Opioid Crisis.

[2] Van Zee A (2009). The promotion and marketing of oxycontin: commercial triumph, public health tragedy. Am J Public Health.

[3] The use of opioids for the treatment of chronic pain (1997). A consensus statement from the American Academy of Pain Medicine and the American Pain Society. Clin J Pain.

[4] Porter J, Jick H (1980). Addiction rare in patients treated with narcotics. N Engl J Med.

[5] Centers for Disease Control and Prevention (2011). Vital signs: overdoses of prescription opioid pain relievers — United States, 1999—2008. MMWR.

[6] Centers for Disease Control and Prevention (2019). 2019 Annual Surveillance Report of Drug-Related Risks and Outcomes—United States. Surveillance Special Report.

[7] Wilson N, Kariisa M, Seth P (2020). Drug and opioid-involved overdose deaths — United States, 2017–2018. MMWR Morb Mortal Wkly Rep.

[8] Butler MM, Ancona RM, Beauchamp GA (2016). Emergency department prescription opioids as an initial exposure preceding addiction. Ann Emerg Med.

[9] Hoppe JA, Kim H, Heard K (2015). Association of emergency department opioid initiation with recurrent opioid use. Ann Emerg Med.

[10] Ward MJ, Kc D, Jenkins CA (2019). Emergency department provider and facility variation in opioid prescriptions for discharged patients. Am J Emerg Med.

[11] Dhalla IA, Mamdani MM, Gomes T (2011). Clustering of opioid prescribing and opioid-related mortality among family physicians in Ontario. Can Fam Physician.

[12] McDonald DC, Carlson K, Izrael D (2012). Geographic variation in opioid prescribing in the U.S.. J Pain.

[13] Smulowitz PB, Cary C, Boyle KL (2016). Variation in opioid prescribing patterns between ED providers. Intern Emerg Med.

[14] Singhal A, Tien YY, Hsia RY (2016). Racial-ethnic disparities in opioid prescriptions at emergency department visits for conditions commonly associated with prescription drug abuse. PLoS One.

[15] Richards LJ, Hopkins NJ, Colwell NA (2019). The association between patient visit demographics and opioid analgesic received in the emergency department. Cureus.

[16] Pletcher MJ, Kertesz SG, Kohn MA (2008). Trends in opioid prescribing by race/ethnicity for patients seeking care in US emergency departments. JAMA.

[17] Weiner SG, Griggs CA, Mitchell PM (2013). Clinician impression versus prescription drug monitoring program criteria in the assessment of drug-seeking behavior in the emergency department. Ann Emerg Med.

[18] Grover CA, Garmel GM (2012). How do emergency physicians interpret prescription narcotic history when assessing patients presenting to the emergency department with pain?. Perm J.

[19] Santoro TN, Santoro JD (2018). Racial bias in the US opioid epidemic: a review of the history of systemic bias and implications for care. Cureus.

[20] Cheatle M, Comer D, Wunsch M (2014). Treating pain in addicted patients: recommendations from an expert panel. Popul Health Manag.

[21] Han B, Compton WM, Jones CM (2015). Nonmedical prescription opioid use and use disorders among adults aged 18 through 64 years in the United States, 2003–2013. JAMA.

[22] Faller RW, Erausquin JT, McCoy TP (2021). Misuse of prescription and illicit drugs in middle adulthood in the context of the opioid epidemic. Subst Use Misuse.

[23] Gladden RM, O’Donnell J, Mattson CL (2019). Changes in opioid-involved overdose deaths by opioid type and presence of benzodiazepines, cocaine, and methamphetamine - 25 States, July–December 2017 to January–June 2018. MMWR Morb Mortal Wkly Rep.

[24] Pike JR, Fadardi JS, Stacy AW (2021). The prospective association between illicit drug use and nonprescription opioid use among vulnerable adolescents. Prev Med.

[25] Abrams DI, Jay CA, Shade SB (2007). Cannabis in painful HIV-associated sensory neuropathy: a randomized placebo-controlled trial. Neurology.

[26] Wilsey B, Marcotte T, Deutsch R (2013). Low-dose vaporized cannabis significantly improves neuropathic pain. J Pain.

[27] Wallace M, Schulteis G, Atkinson JH (2007). Dose-dependent effects of smoked cannabis on capsaicin-induced pain and hyperalgesia in healthy volunteers. Anesthesiology.

[28] Everson EM, Dilley JA, Maher JE (2019). Post-legalization opening of retail cannabis stores and adult cannabis use in Washington State, 2009–2016. Am J Public Health.

[29] Chihuri S, Li G (2019). State marijuana laws and opioid overdose mortality. Inj Epidemiol.

[30] Liang D, Bao Y, Wallace M (2018). Medical cannabis legalization and opioid prescriptions: evidence on US Medicaid enrollees during 1993–2014. Addiction.

[31] Shi Y, Liang D, Bao Y (2019). Recreational marijuana legalization and prescription opioids received by Medicaid enrollees. Drug Alcohol Depend.

[32] Nielsen S, Sabioni P, Trigo JM (2017). Opioid-sparing effect of cannabinoids: a systematic review and meta-analysis. Neuropsychopharmacology.

[33] Bahji A, Hargreaves T, Finch S (2018). Assessing the utility of drug screening in the emergency: a short report. BMJ Open Qual.

[34] Becerra X (2018). State of California department of justice.

[35] Wen H, Hockenberry JM (2018). Association of medical and adult-use marijuana laws with opioid prescribing for Medicaid enrollees. JAMA Intern Med.

[36] Olfson M, Wall MM, Liu SM (2018). Cannabis use and risk of prescription opioid use disorder in the United States. Am J Psychiatry.

[37] DiBenedetto D, Weed VF, Wawrzyniak KM (2018). The association between cannabis use and aberrant behaviors during chronic opioid therapy for chronic pain. Pain Med.

[38] Bhashyam AR, Heng M, Harris MB (2018). Self-reported marijuana use is associated with increased use of prescription opioids following traumatic musculoskeletal injury. J Bone Joint Surg Am.

[39] Liang D, Wallace MS, Shi Y (2019). Medical and non-medical cannabis use and risk of prescription opioid use disorder: Findings from propensity score matching. Drug Alcohol Rev.

[40] (2018). AB-2760 Prescription drugs: prescribers: naloxone hydrochloride and other FDA-approved drugs 2017–2018.

[41] (2014). Premature Mortality in Orange County.

[42] Chandler J (2016). Fatal drug overdoses reach 10 year high in Orange County. OC Register.

[43] Dwyer KH, Samuels L, Moore RL (2013). Physician attitudes and perceived barriers to prescribing nasal naloxone rescue kits in the emergency department. Ann Emerg Med.

[44] Marino R, Landau A, Lynch M (2019). Do electronic health record prompts increase take-home naloxone administration for emergency department patients after an opioid overdose?. Addiction.

[45] California Proposition 64. Marijuana Legalization Initiative Statute.

