# Supplementary material for: Positive Toxicology Results Are Not Associated with Emergency Physicians’ Opioid Prescribing Behavior
Source: West J Emerg Med. 2021 Aug 30;22(5):1067–75. doi: 10.5811/westjem.2021.5.52378 (PMC8463062; doi:10.5811/westjem.2021.5.52378)
Supplement: Supplementary file 1 [file wjem-22-1067-s001.docx]

Table 1: Comparison of opioid prescriptions between THC+, THC-, and no toxicology screens

|  | Presence of THC | Statistic | Value |
| --- | --- | --- | --- |
| Total Prescribed MME | Negative | Mean | 105.0 |
|  |  | Median | 75.0 |
|  |  | Std. Deviation | 82.72 |
|  |  | Minimum | 30.0 |
|  |  | Maximum | 540.0 |
|  | Positive | Mean | 157.1 |
|  |  | Median | 87.5 |
|  |  | Std. Deviation | 337.91 |
|  |  | Minimum | 50.0 |
|  |  | Maximum | 1800.0 |
|  | No Urine or Serum Toxicology Obtained | Mean | 90.0 |
|  |  | Median | 75.0 |
|  |  | Std. Deviation | 74.69 |
|  |  | Minimum | 15.0 |
|  |  | Maximum | 900.0 |
| Amount prescribed (liquids are divided by concentration) | Negative | Mean | 16.6 |
|  |  | Median | 15.0 |
|  |  | Std. Deviation | 9.54 |
|  |  | Minimum | 6.0 |
|  |  | Maximum | 72.0 |
|  | Positive | Mean | 18.2 |
|  |  | Median | 15.0 |
|  |  | Std. Deviation | 11.16 |
|  |  | Minimum | 10.0 |
|  |  | Maximum | 60.0 |
|  | No Urine or Serum Toxicology Obtained | Mean | 15.3 |
|  |  | Median | 15.0 |
|  |  | Std. Deviation | 7.27 |
|  |  | Minimum | 3.0 |
|  |  | Maximum | 60.0 |
